# Baicalin potentiates cefquinome against animal-origin ESBL-*Escherichia coli* by attenuating resistance-associated phenotypes and intestinal injury

**DOI:** 10.3389/fvets.2026.1869565

**Published:** 2026-07-31

**Authors:** Yu-Han Jiang, Lei-Xin Zhu, Zi-Xu Cheng, Shao-Qiang Sun, Wei-Min Zhang, Su-Zhu Qing

**Affiliations:** College of Veterinary Medicine, Northwest A&F University, Yangling, China

**Keywords:** antibiotic adjuvant, baicalin, cefquinome, ESBL-*E. coli*, gut microbiota, intestinal injury

## Abstract

Animal-origin extended-spectrum *β*-lactamase-producing *Escherichia coli* (ESBL-*E. coli*) undermines cefquinome (CEF) efficacy in veterinary medicine, necessitating strategies that enhance antibacterial activity while protecting intestinal integrity. Here, we evaluated baicalin (BAI), a flavonoid from *Scutellaria baicalensis*, as a CEF adjuvant against animal-origin ESBL-*E. coli*. In a mouse intestinal infection model, BAI combined with CEF significantly improved survival, mitigated intestinal pathology, and downregulated *Tlr4*, *Myd88*, and NF-κB signaling. The combination preserved mucosal architecture and partially restored intestinal microbiota composition. *In vitro*, BAI potentiated CEF activity by suppressing *blaCTX-M* gene expression, reducing ESBL-mediated CEF hydrolysis, and limiting the emergence of reduced CEF susceptibility during serial passage. Docking suggested a potential interaction of BAI with the catalytic site of CTX-M-9; together with reduced cefquinome hydrolysis in whole-cell suspensions, this suggests interference with ESBL-mediated hydrolysis. These results demonstrate that BAI functions as a CEF sensitizer, integrating pathogen-directed and host-protective effects, and offer a translationally relevant strategy for veterinary antibiotic adjuvant therapy.

## 1 Introduction

Animal-origin extended-spectrum beta-lactamase (ESBL)-producing *Escherichia coli* (ESBL-*E. coli*) has become an increasing challenge in veterinary medicine because of its pathogenicity, resistance burden, and dissemination potential (1, 2). As important reservoirs of mobile beta-lactam resistance determinants, particularly *blaCTX-M*-type genes, ESBL-*E. coli* substantially compromises the clinical efficacy of cephalosporins such as cefquinome (CEF) and complicates anti-infective treatment in animals (3). CEF is a fourth-generation cephalosporin used exclusively in veterinary medicine (4). Therefore, strategies that restore or preserve CEF activity against animal-origin ESBL-producing *E. coli* are directly relevant to veterinary antimicrobial stewardship (5).

In intestinal infection, therapeutic success depends on more than bacterial clearance alone. Infection-driven inflammation disrupts epithelial integrity, impairs mucosal barrier function, and reshapes the intestinal microbial community, thereby aggravating tissue injury and delaying recovery (6, 7). An effective therapeutic strategy should therefore not only suppress the pathogen, but also mitigate infection-associated intestinal damage (8).

Baicalin (BAI), a major flavonoid derived from *Scutellaria baicalensis*, is of particular interest in this context because it has been reported to possess antibacterial, anti-inflammatory, and barrier-protective activities (9–11). In addition, growing evidence suggests that BAI or related flavonoids can enhance antibiotic activity against resistant bacteria and interfere with resistance-associated phenotypes (12, 13). These properties raise the possibility that BAI may function not simply as a direct antimicrobial compound, but as an antibiotic adjuvant that links pathogen sensitization with host protection (14).

However, current evidence for BAI as an antibiotic adjuvant remains focused mainly on *in vitro* antibacterial interaction, whereas its contribution within the infection-disrupted intestinal microenvironment is still poorly defined. In particular, it remains unclear whether BAI can improve CEF efficacy against animal-origin ESBL-*E. coli* through simultaneous attenuation of bacterial resistance-associated phenotypes and mitigation of infection-induced intestinal injury (13, 15).

To address this question, we evaluated the therapeutic efficacy of BAI combined with CEF in a mouse challenge model of intestinal injury induced by animal-origin ESBL-*E. coli*. We further examined its effects on intestinal inflammatory signaling, mucosal barrier-related indices, and intestinal microbiota composition, while also investigating *in vitro* antibacterial interaction, CEF hydrolysis, *blaCTX-M* expression, resistance development, and molecular docking of BAI with the CTX-M-9 *β*-lactamase catalytic pocket. This study was designed to determine whether BAI can act as a CEF adjuvant in veterinary anti-infective therapy by combining pathogen-directed and host-protective effects. Unlike studies focusing solely on *in vitro* antibacterial synergy, this study integrates bacterial resistance-associated phenotypes with host intestinal injury, microbiota responses, and potential molecular interactions, thereby providing a more infection-relevant evaluation of BAI as a veterinary antibiotic adjuvant.

## 2 Materials and methods

### 2.1 Bacterial strains and culture conditions

The reference strain *E. coli* ATCC 25922 and 3 animal-origin ESBL-producing isolates were used. ZZ8 was selected for the mouse challenge model based on stable ESBL phenotype and CEF resistance. Frozen stocks were recovered on Mueller–Hinton agar at 37 °C for 12–16 h. Single colonies were inoculated into Mueller–Hinton broth and grown with shaking to logarithmic phase. Bacterial suspensions were adjusted by optical density and confirmed by viable colony counting when necessary.

### 2.2 Animals and experimental design

All animal procedures were reviewed and approved by the Institutional Animal Care and Use Committee of Northwest A&F University, China (Approval No. NWLA-2021-063), and were conducted in accordance with institutional guidelines for animal care and use.

Ninety-six 8-week-old specific pathogen-free female Kunming mice weighing 20–22 g were obtained from Beijing Vital River Laboratory Animal Technology Co., Ltd. (Beijing, China). Mice were housed under standard laboratory conditions at 22 ± 2 °C and 50 ± 10% relative humidity under a 12 h light/dark cycle, with free access to food and water. After acclimatization, mice were randomly assigned to experimental groups. Histological and morphometric analyses were performed in a blinded manner to minimize observational bias.

A mouse model of ESBL-*E. coli*-associated intestinal inflammatory injury was established using a combined intraperitoneal and oral challenge with ESBL-producing *E. coli* ZZ8 (16, 17). This model was designed to induce a reproducible acute infection-associated intestinal injury condition for evaluating the therapeutic efficacy and intestinal protective effects of BAI and CEF.

Three consecutive animal experiments were performed.

#### 2.2.1 Experiment 1: LD50 determination

Twenty-four mice were randomly divided into four groups, with six mice in each group. Three groups were challenged with different concentrations of ESBL-producing *E. coli* ZZ8, whereas the control group received sterile saline. Mortality and clinical signs were monitored after challenge, and the median lethal dose was determined according to the observed mortality.

#### 2.2.2 Experiment 2: evaluation of therapeutic efficacy

Thirty-six mice were randomly divided into six groups: normal control group, model group, CEF group, BAI group, low-dose combination group, and high-dose combination group. Except for the normal control group, all mice were challenged with ESBL-producing *E. coli* ZZ8 at the predetermined LD50 dose. Drug treatment was initiated 3 h after bacterial challenge and continued once daily for three consecutive days according to the regimen shown in Table 1. Survival, clinical signs, and general health status were monitored during the observation period.

**Table 1:** Experimental groups, bacterial challenge, and treatment regimens in the mouse infection model.

Group	Challenge inoculum	Treatment regimen
Negative control (NC)	10 μL/g physiological saline	10 μL/g physiological saline
Model control (MOD)	10 μL/g of ESBL-*E. coli* strain ZZ8 suspension (1 × 10^9^ CFU/mL)	10 μL/g physiological saline
Cefquinome (CEF)	10 μL/g of ESBL-*E. coli* strain ZZ8 suspension (1 × 10^9^ CFU/mL)	5 mg/kg Cefquinome
Baicalin (BAI)	10 μL/g of ESBL-*E. coli* strain ZZ8 suspension (1 × 10^9^ CFU/mL)	5 mg/kg Baicalin
Low-dose Combination (COM. L)	10 μL/g of ESBL-*E. coli* strain ZZ8 suspension (1 × 10^9^ CFU/mL)	5/4 mg/kg Cefquinome & 5/2 mg/kg Baicalin
High-dose Combination (COM. H)	10 μL/g of ESBL-*E. coli* strain ZZ8 suspension (1 × 10^9^ CFU/mL)	5/2 mg/kg Cefquinome & 5/2 mg/kg Baicalin

#### 2.2.3 Experiment 3: evaluation of therapeutic mechanisms

Thirty-six mice were randomly assigned to the same six groups described above. The bacterial challenge and treatment protocols were identical to those used in Experiment 2. At 24 h after challenge, mice were anesthetized with isoflurane inhalation and euthanized by cervical dislocation for sample collection.

Jejunal tissues were collected for histopathological examination, quantitative real-time PCR, and estimation of tissue-associated bacterial burden. Duodenal, jejunal, and ileal segments were collected for intestinal morphometric and cellular analyses. Ileal tissues were collected for secretory immunoglobulin A detection. Cecal contents were collected under sterile conditions and stored at −80 °C until microbiota analysis.

### 2.3 Histopathology and intestinal morphometry

Intestinal tissue samples were fixed in 4% paraformaldehyde, embedded in paraffin, and sectioned at 5 μm. Sections were stained with hematoxylin–eosin (HE) to evaluate general morphology and with periodic acid–Schiff (PAS) to visualize goblet cells, following standard histological protocols (18). Morphometric parameters including villus height, crypt depth, and villus/crypt ratio were measured using ImageJ software from at least five well-oriented villus–crypt units per intestinal segment (19). PAS-positive goblet cells and intraepithelial lymphocytes were counted in five randomly selected villi per segment. All observations and measurements were performed blinded, and representative images were captured from comparable regions across groups.

### 2.4 Quantitative real-time PCR (qPCR)

Total RNA was extracted from bacterial cultures and intestinal tissues using TRIzol reagent according to the manufacturer’s instructions. Briefly, logarithmic-phase bacterial cultures or intestinal tissues were homogenized in TRIzol, followed by chloroform extraction and isopropanol precipitation. RNA concentration and purity were assessed using a NanoDrop spectrophotometer, and qualified RNA samples were reverse-transcribed into cDNA using a reverse transcription kit with genomic DNA removal.

Quantitative real-time PCR was performed using SYBR Green Master Mix in a 20 μL reaction system. The amplification conditions were 95 °C for 3 min, followed by 40 cycles of 95 °C for 10 s and 60 °C for 30 s, with melting curve analysis conducted to verify amplification specificity. The 16S rRNA gene and *β*-actin were used as internal controls for bacterial and intestinal tissue samples, respectively. Relative gene expression was calculated using the 2^-ΔΔCt method (20, 21). Primer sequences are listed in Table 2. All reactions were performed in triplicate, and no-template controls were included to exclude contamination.

**Table 2:** Primer sequences used for quantitative PCR.

Gene	Forward primer (5′ ~ 3′)	Reverse primer (5′ ~ 3′)
*16S rRNA*	AGAGTTTGATCCTGGCTCAG	CTTGTGCGGGCCCCCGTCAATTC
*bla*_CTX-M-9_	GCACGATGACATTCGGG	AACCCACGATGTGGGTAGC
*Tlr4*	TCAGCAAAGTCCCTGATGAC	TGCCATGCCTTGTCTTCAAT
*Myd88*	CCTGAGTCCCCAAGAAAGTG	GTACAAACACGAGCCCTTCT
*Nfkb1*	TCTGGCGAATGGCTTTACTT	GGGTGTGTCTTGGTGGTATC
*β-actin*	ATCACTATTGGCAACGAGCGGTTC	CAGCACTGTGTTGGCATAGAGGTC

### 2.5 ELISA detection of ileal secretory immunoglobulin a

Ileal tissue samples were homogenized in cold phosphate-buffered saline and centrifuged to collect the supernatant. Secretory immunoglobulin A concentration was measured using a commercial mouse SIgA ELISA kit (Shanghai Enzyme-linked Biotechnology Co., Ltd., China) according to the manufacturer’s instructions. Absorbance was measured at 450 nm using a microplate reader, and sample concentrations were calculated from the standard curve.

### 2.6 Intestinal microbiota analysis

Cecal contents were collected under sterile conditions and stored at −80 °C until DNA extraction. Microbial genomic DNA was extracted, and DNA quality was verified before amplification. The V3–V4 hypervariable region of the bacterial 16S rRNA gene was amplified using primers 338F 5′-CCTAYGGGRBGCASCAG-3′ and 806R 5′-GGACTACNNGGGTATCTAAT-3′. Purified amplicons were sequenced on an Illumina NovaSeq 6,000 platform using paired-end sequencing. Raw reads were quality-filtered using fastp v0.23.1. Amplicon sequence variants were generated using the DADA2 pipeline in QIIME2 version 2022.02. Taxonomic annotation was performed using the SILVA 138.1 database. Alpha diversity was evaluated using Chao1, Shannon, and Simpson indices. Beta diversity was assessed by principal coordinate analysis based on Bray–Curtis distances. Differences in microbial community structure among groups were evaluated using PERMANOVA. Differentially abundant taxa were identified using the Kruskal–Wallis test followed by Benjamini–Hochberg correction. Adjusted *p* values < 0.05 were considered statistically significant (21–23).

### 2.7 *In vitro* antibacterial assays and time-kill experiments

The MICs of BAI and CEF were determined in Mueller–Hinton broth using standard broth microdilution, to guide dosing for time-kill experiments. Logarithmic-phase ESBL-producing *E. coli* cultures were diluted into fresh broth and allocated to the following groups: drug-free control, CEF alone, BAI alone, low-dose combination, and high-dose combination. Cultures were incubated at 37 °C, and aliquots were collected at 0, 1, 2, 4, 6, 12, and 24 h. Samples were serially diluted, plated on Mueller–Hinton agar, and incubated for colony counting (24–26).

### 2.8 Resistance development assay

The influence of BAI on the emergence of reduced CEF susceptibility was assessed via serial passage under subinhibitory drug exposure. Logarithmic-phase ESBL-producing *E. coli* cultures were diluted into fresh Mueller–Hinton broth containing CEF alone, CEF combined with BAI, or no drug (control). Cultures were incubated at 37 °C for 24 h, after which CEF MICs were determined by standard broth microdilution. Cultures were then transferred into fresh medium with the corresponding drug condition for the next passage. This procedure was repeated for 15 consecutive passages, with MICs recorded at passages 0, 1, 2, 3, 6, 9, and 15. All assays were performed in triplicate to ensure reproducibility (27, 28).

### 2.9 CEF hydrolysis assay

BAI’s effect on CEF hydrolysis by ESBL-producing *E. coli* was evaluated using a cephalosporin hydrolysis assay. Logarithmic-phase cultures of strains ZZ4, ZZ8, and ZZ9 were incubated with or without BAI. Cells were harvested, washed, and resuspended in Tris–HCl buffer, then incubated with CEF at 37 °C. The reaction was terminated by adding hydrochloric acid followed by boiling to convert CEF into its anhydrous form. After centrifugation, the absorbance of the supernatant at 270 nm was measured. Residual CEF concentrations were calculated using a standard curve, with higher residual levels indicating reduced CEF hydrolysis. As whole bacterial suspensions were used rather than purified *β*-lactamase, the results reflect the *β*-lactamase-associated hydrolytic phenotype rather than direct enzymatic inhibition.

### 2.10 Molecular docking

Molecular docking was performed to explore potential interactions between BAI and CTX-M-9 β-lactamase. The three-dimensional structure of BAI was obtained from PubChem, and the CTX-M-9 structure from a public protein database. Protein preparation involved removal of water molecules and irrelevant ligands, hydrogen addition, charge assignment, and energy minimization. Ligand preparation included geometry optimization and charge assignment. Docking was conducted using AutoDock Vina, with a grid covering the catalytic pocket of CTX-M-9. The lowest-energy docking pose with appropriate orientation within the catalytic pocket was selected and visualized. Docking scores were interpreted as computational evidence supporting potential binding compatibility between BAI and CTX-M-9, rather than direct inhibition (29).

### 2.11 Statistical analysis

Data are presented as mean ± SD. Multiple-group comparisons were analyzed by one-way ANOVA followed by Tukey’s *post hoc* test; two-group comparisons by Student’s t-test. Survival curves were plotted using Kaplan–Meier analysis and compared by log-rank test. Microbiota beta-diversity differences were assessed via PERMANOVA, and differential taxa identified using Kruskal–Wallis test with Benjamini–Hochberg correction for false discovery rate. *p* < 0.05 was considered statistically significant. GraphPad Prism 9.5 and SPSS 20.0 were used for all analyses.

## 3 Results

### 3.1 BAI enhances the therapeutic efficacy of CEF against ESBL-*E. coli* in mice

**Figure 1:**
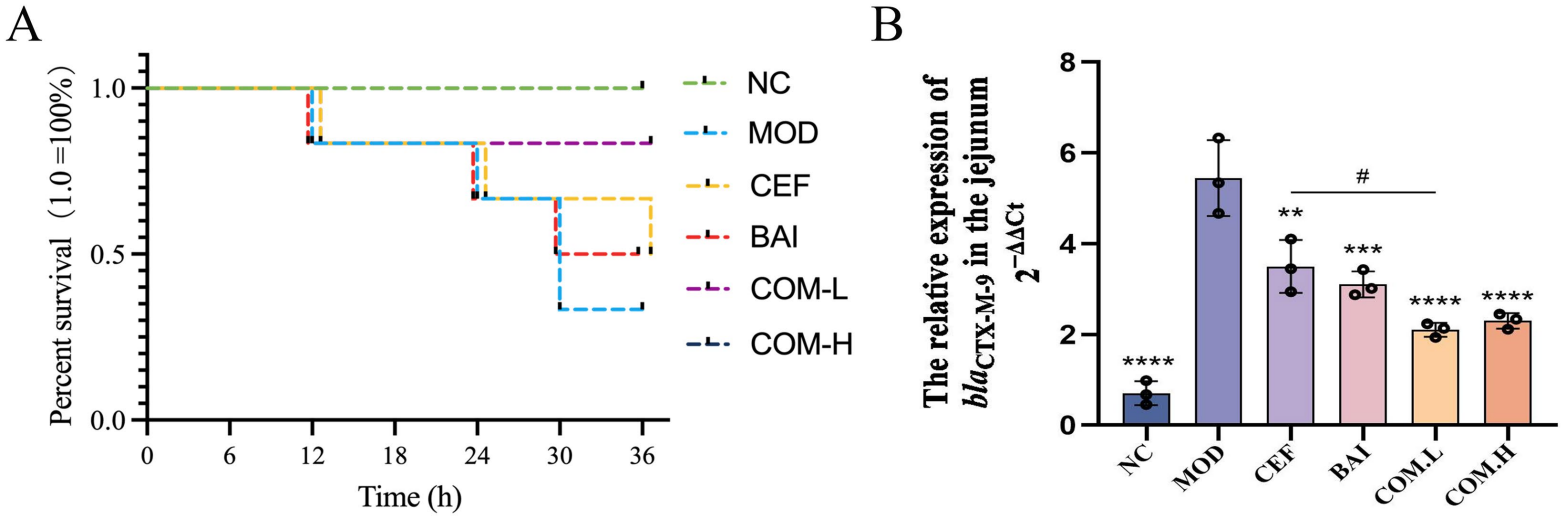
Therapeutic efficacy of baicalin (BAI) in combination with cefquinome (CEF) against ESBL-producing *E. coli* ZZ8 in mice. **(A)** Survival curves of mice in different treatment groups during 24 h post-challenge: Normal control (NC), model (MOD), CEF monotherapy, BAI monotherapy, low-dose combination (COM. L), and high-dose combination (COM. H). **(B)** Jejunal bacterial burden measured as relative *blaCTX-M-9*-associated signal by qPCR. Data are presented as mean ± SD (*n* = 6 per group). Statistical significance was determined using one-way ANOVA with Tukey’s *post hoc* test; *p* < 0.05 compared with MOD group.

To evaluate whether BAI potentiates CEF *in vivo*, we assessed survival, clinical manifestations, and intestinal bacterial burden in mice challenged with ESBL-producing *E. coli* ZZ8. In the infection model, the MOD group exhibited severe disease, including depressed mental status, reduced food and water intake, huddling for warmth at 36 h, and severe diarrhea, resulting in a 67% mortality rate. CEF monotherapy partially protected mice, reducing mortality to 50%, but clinical signs such as depression, poor intake, and severe diarrhea persisted, indicating that antibacterial treatment alone was insufficient to fully reverse disease severity. Similarly, BAI monotherapy decreased mortality to 50% and moderately alleviated diarrhea, but mental status remained depressed. Combination therapy produced more pronounced benefits. The low-dose combination (COM. L) reduced mortality to 16%, partially restored behavior, and shortened recovery time, with 16% of mice regaining normal activity by 24 h. The high-dose combination (COM. H) achieved complete survival (0% mortality), rapid recovery of mental status within 24 h, mild diarrhea, and 50% of mice regained normal activity within the first day post-challenge (Figure 1A).

To quantify bacterial burden, jejunal blaCTX-M-9-associated signals were measured by qPCR. Compared with the MOD group, both combination groups exhibited significantly reduced blaCTX-M-9 levels, with COM. H showing the lowest bacterial signal, followed by COM. L. BAI monotherapy also reduced jejunal bacterial burden relative to MOD, although to a lesser extent than the combination treatments (Figure 1B).

These findings demonstrate that BAI enhances CEF therapeutic efficacy by improving survival, mitigating clinical severity, and reducing intestinal bacterial load. The effect is dose-dependent, with the high-dose combination providing the most complete protection, and the low-dose combination achieving a balanced improvement in both survival and recovery indicators.

### 3.2 BAI alleviates ESBL-*E. coli*-induced intestinal inflammatory and structural injury during CEF treatment

**Figure 2:**
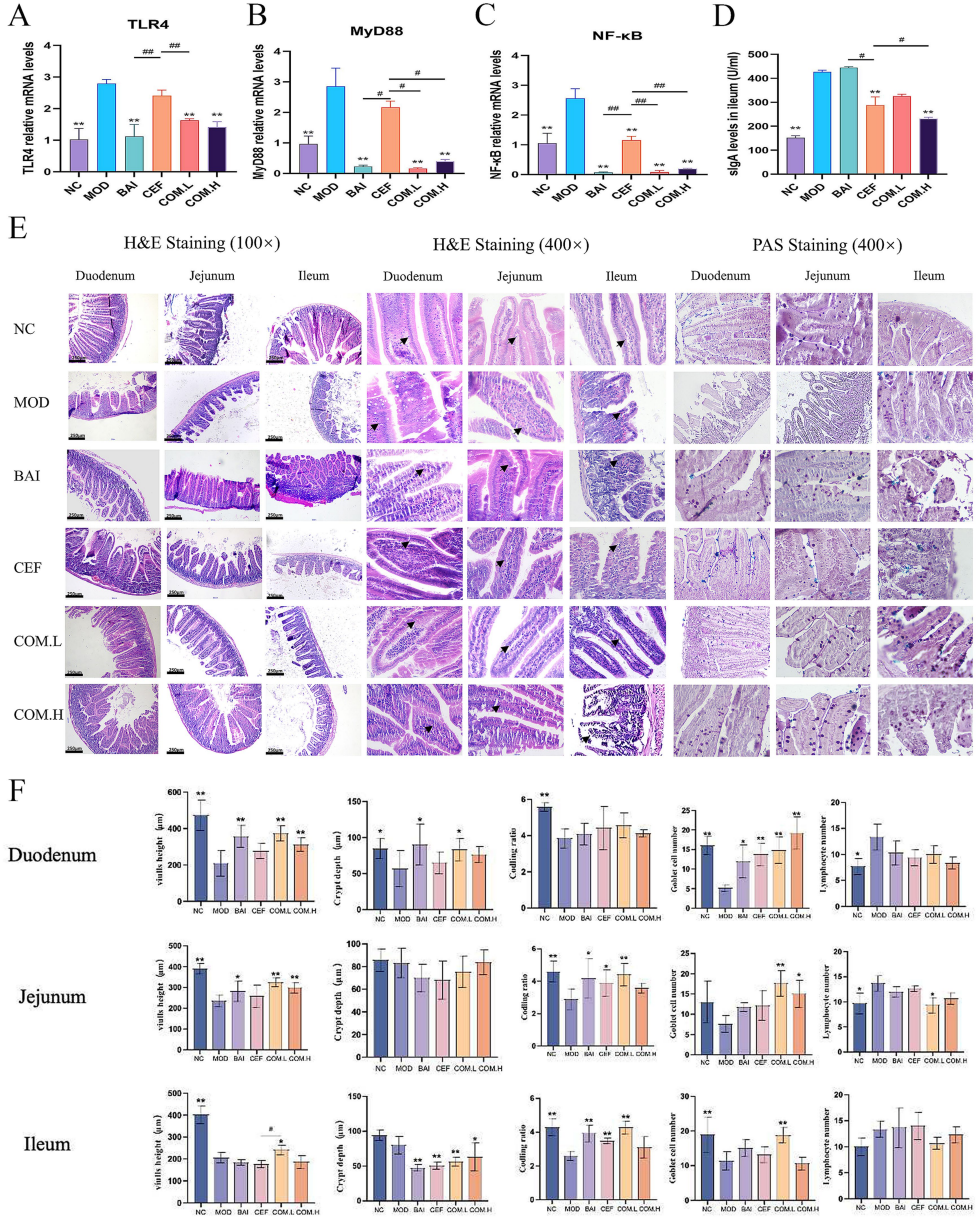
Baicalin (BAI) alleviates intestinal inflammatory and structural injury induced by ESBL-producing *E. coli* during cefquinome (CEF) treatment in mice. **(A–C)** Relative mRNA expression levels of jejunal *Tlr4*, *Myd88*, and NF-κB-associated inflammatory genes in different treatment groups (NC, MOD, CEF, BAI, COM. L, COM. H), measured by qPCR. **(D)** Ileal secretory immunoglobulin A (sIgA) concentration assessed by ELISA. **(E)** Representative hematoxylin and eosin-stained jejunal sections showing villus structure and inflammatory cell infiltration (scale bar = 50 μm). **(F)** Quantitative morphometric analysis of villus height/crypt depth ratio, goblet cell count, and intraepithelial lymphocyte number. Data are presented as mean ± SD (*n* = 6). Statistical significance determined using one-way ANOVA with Tukey’s post hoc test; *p* < 0.05 versus MOD group.

We next investigated whether the improved therapeutic efficacy was accompanied by attenuation of intestinal injury. ESBL-*E. coli* challenge induced marked inflammatory activation in jejunal tissue, as reflected by significant upregulation of *Tlr4*, *Myd88*, and NF-κB-related genes in the MOD group compared with the NC group. CEF monotherapy did not markedly reduce this inflammatory response, whereas BAI-containing treatments significantly downregulated all three genes. In addition, expression levels in the BAI, COM. L, and COM. H groups were lower than those in the CEF group, indicating that BAI contributed substantially to suppression of infection-associated intestinal inflammation (Figures 2A–C).

We then evaluated mucosal immune response by measuring ileal secretory immunoglobulin A (sIgA). ESBL-*E. coli* challenge significantly increased sIgA levels relative to the NC group, consistent with activation of mucosal immune defense after infection. Compared with the MOD group, sIgA levels were reduced in the CEF and COM. H groups, whereas BAI monotherapy showed an opposite trend and maintained relatively higher sIgA levels. These findings indicate that sIgA levels changed in a treatment-dependent manner under infection conditions, but the immunological mechanisms underlying these changes require further validation (Figure 2D).

Histopathological evaluation further showed that infection caused marked intestinal injury, including villus destruction, epithelial damage, lamina propria disruption, edema, and inflammatory cell infiltration. Although all treatment groups exhibited some degree of histological improvement, BAI-containing regimens more effectively preserved tissue architecture than CEF alone (Figure 2E). Quantitative morphometric analysis confirmed this pattern. In the duodenum and jejunum, villus height was significantly increased in BAI-treated and combination-treated groups relative to the MOD group. Among these regimens, the low-dose combination showed the most consistent improvement across intestinal segments and maintained a more favorable villus height/crypt depth ratio. In the ileum, the protective effect was also most evident in the COM. L group (Figure 2F).

Goblet cell analysis revealed segment-specific responses to infection and treatment. Infection decreased goblet cell abundance, particularly in the ileum, whereas combination treatment promoted goblet cell recovery. In the jejunum, both combination groups restored goblet cell numbers relative to the MOD group. In the ileum, this protective effect was most evident in the COM. L group. Intraepithelial lymphocyte counts showed an opposite pattern: infection increased inflammatory cell infiltration, particularly in the duodenum and jejunum, whereas combination treatment, especially COM. L, reduced this response (Figure 2F).

Taken together, these findings indicate that BAI contributes to CEF efficacy not only by improving overall therapeutic outcome, but also by alleviating ESBL-*E. coli*-induced intestinal inflammatory and structural injury. Among the tested regimens, the low-dose combination showed the most balanced protective effect on intestinal architecture and local inflammatory status.

### 3.3 BAI partially restores intestinal microbiota homeostasis under ESBL-*E. coli* challenge

**Figure 3:**
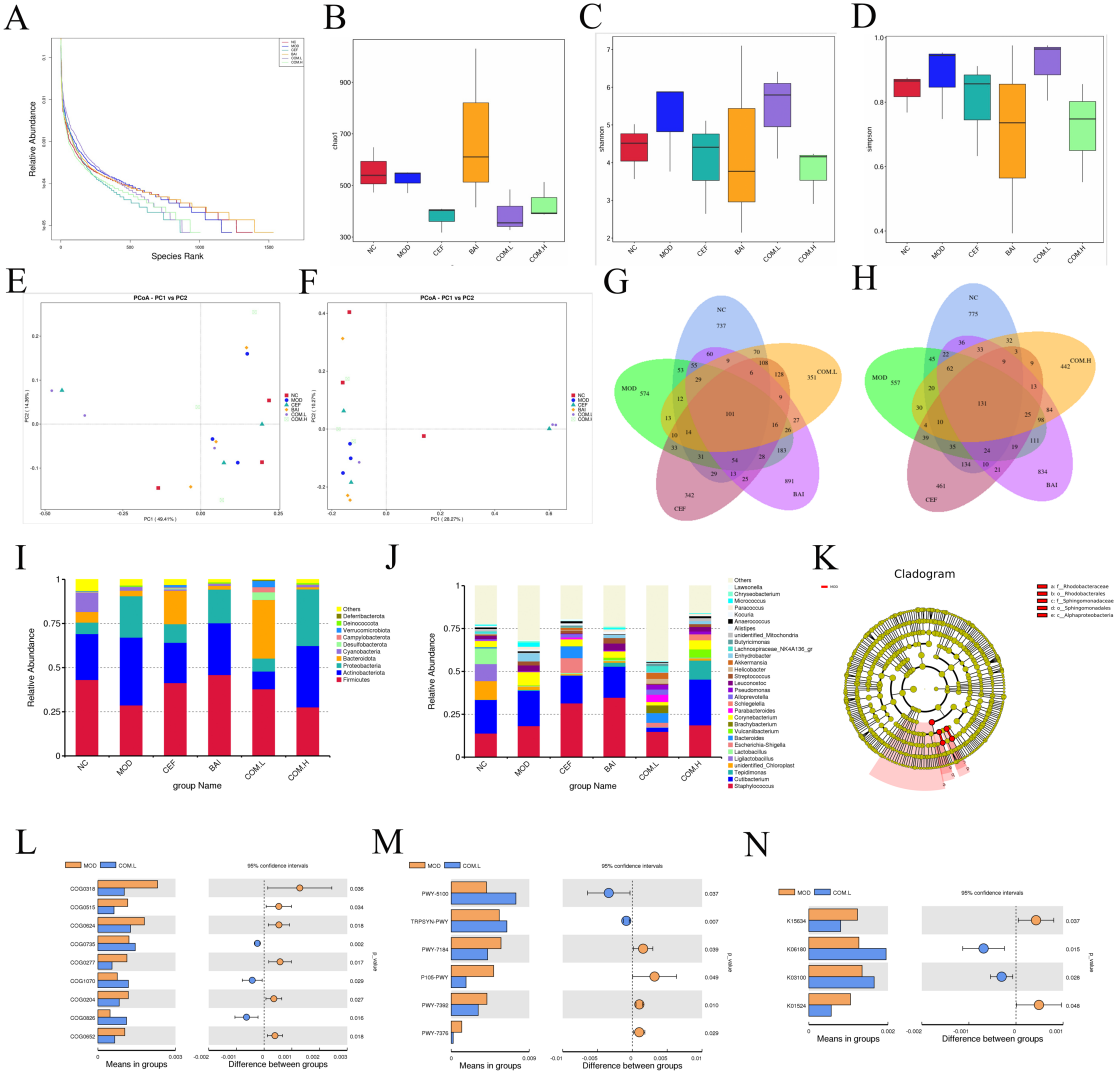
Baicalin (BAI) partially restores intestinal microbiota homeostasis under ESBL-producing *E. coli* challenge in mice. **(A)** Rank-abundance curves showing microbial richness and evenness in different treatment groups (NC, MOD, CEF, BAI, COM. L, COM. H). **(B–D)** Alpha-diversity indices: Chao1, Shannon, and Simpson. **(E,F)** Principal coordinate analysis (PCoA) of beta-diversity based on Bray–Curtis distances. **(G,H)** Venn diagrams of shared and unique amplicon sequence variants (ASVs) among groups. **(I,J)** Taxonomic composition at phylum and genus levels. **(K)** LEfSe cladogram indicating differentially abundant taxa contributing to intergroup differences. Data are presented as mean ± SD (*n* = 6). Statistical significance determined using Kruskal–Wallis test followed by Benjamini–Hochberg correction; *p* < 0.05 versus MOD group. **(L–N)** PICRUSt2-based functional prediction of gut microbiota combined with annotations.

Because intestinal injury is closely associated with microbial dysbiosis, we next investigated whether BAI-containing treatments affected the intestinal microbiota under ESBL-*E. coli* challenge. Rank-abundance analysis showed that all groups maintained relatively broad microbial richness and community evenness, although differences in curve distribution suggested treatment-related variation in community composition (Figure 3A).

Alpha-diversity analysis was performed using Chao1, Shannon, and Simpson indices to evaluate microbial richness, overall diversity, and community evenness, respectively. Compared with the MOD group, the BAI group showed a higher Chao1 index, indicating a numerical increase in microbial richness, whereas the COM. L and COM. H groups showed relatively lower richness values (Figure 3B). The Shannon and Simpson indices also varied among groups, with the COM. L group showing relatively higher diversity and evenness, while the BAI and COM. H groups displayed lower or intermediate values (Figures 3C,D). These findings suggest that ESBL-*E. coli* challenge and subsequent treatments influenced within-sample microbial diversity, although the direction of change differed among BAI monotherapy and combination regimens.

Beta-diversity analysis based on principal coordinate analysis revealed distinct shifts in overall cecal microbial community structure among groups (Figures 3E,F). The MOD group was separated from the NC group, indicating that ESBL-*E. coli* challenge altered the cecal microbial community. Notably, samples from BAI-containing treatment groups showed a shift away from the MOD group and partially overlapped with or approached the NC distribution, suggesting that BAI intervention contributed to partial remodeling of infection-disrupted microbiota structure.

Venn analysis further showed that the six groups shared a core set of ASVs, while each group also contained group-specific ASVs (Figures 3G,H). The presence of both shared and unique ASVs indicates that ESBL-*E. coli* challenge and drug intervention reshaped the cecal microbial community at the ASV level. In particular, BAI-containing groups displayed distinct ASV profiles compared with the MOD group, supporting treatment-associated microbial remodeling rather than a simple restoration of the original community.

Taxonomic composition analysis further supported these community-level changes. At the phylum level, Firmicutes, Actinobacteriota, Proteobacteria, Bacteroidota, Campylobacterota, and Desulfobacterota were the dominant bacterial phyla across groups (Figure 3I). Compared with the NC group, the MOD group showed a marked reduction in Firmicutes and an increased relative abundance of Actinobacteriota and Bacteroidota, indicating infection-associated dysbiosis. BAI treatment increased the relative abundance of Firmicutes and reduced the relative abundance of Bacteroidota compared with the MOD group. The combination groups also showed distinct phylum-level redistribution, suggesting that BAI and CEF treatment reshaped the infection-altered microbial structure.

At the genus level, the dominant genera included Staphylococcus, Cutibacterium, Tepidimonas, unidentified Chloroplast, Lactobacillus, Escherichia-Shigella, Bacteroides, Brachybacterium, Corynebacterium, and Parabacteroides (Figure 3J). ESBL-*E. coli* challenge altered the relative abundance of several dominant genera, whereas BAI-containing treatments produced distinct genus-level profiles. These changes indicate that BAI treatment was associated with taxonomic redistribution of the intestinal microbiota under infection conditions.

LEfSe cladogram analysis identified several bacterial taxa that contributed to intergroup differences (Figure 3K). The MOD group was enriched in taxa belonging to Rhodobacteraceae, Rhodobacterales, Alphaproteobacteria, and related lineages, suggesting that ESBL-*E. coli* challenge induced specific microbial signatures. These differential taxa further support the presence of infection-associated microbial disturbance and treatment-related community remodeling.

To further assess whether microbiota remodeling was accompanied by functional changes, PICRUSt2 was used to infer microbial functional profiles. At the COG level, COG0318, COG0515, COG0624, COG0277, COG0204, and COG0652 were significantly enriched in the MOD group compared with the COM. L group, whereas COG0735, COG1070, and COG0826 were enriched in the COM. L group. MetaCyc pathway prediction showed higher abundances of PWY-7184, P105-PWY, PWY-7392, and PWY-7376 in the MOD group, while PWY-5100 and TRPSYN-PWY were increased in the COM. L group. Consistently, KEGG orthology analysis identified K15634 and K01524 as MOD-enriched functions, whereas K06180 and K03100 were enriched in COM. L. Collectively, these results indicate that COM. L treatment reshaped the predicted microbial functional profile, with reduced model-associated enrichment of energy metabolism, nucleotide/cofactor biosynthesis, and membrane/lipid-related functions, together with increased predicted potential for fermentation, tryptophan biosynthesis, protein processing, RNA modification, and iron-homeostasis regulation (Figures 3L,N).

Together, these results indicate that ESBL-*E. coli* challenge disturbed the cecal microbial community, while BAI-containing treatments partially reshaped microbiota composition at the alpha-diversity, beta-diversity, ASV, phylum, and genus levels. Although these findings do not establish a causal role for specific taxa, they suggest that the intestinal protective effect of BAI may be associated with partial restoration of cecal microbial homeostasis under ESBL-*E. coli* challenge.

### 3.4 BAI potentiates CEF *in vitro* by attenuating resistance-associated phenotypes in ESBL-*E. coli*

To investigate the pathogen-directed basis for the *in vivo* beneficial effect of BAI, its interaction with CEF was examined in vitro.

**Figure 4:**
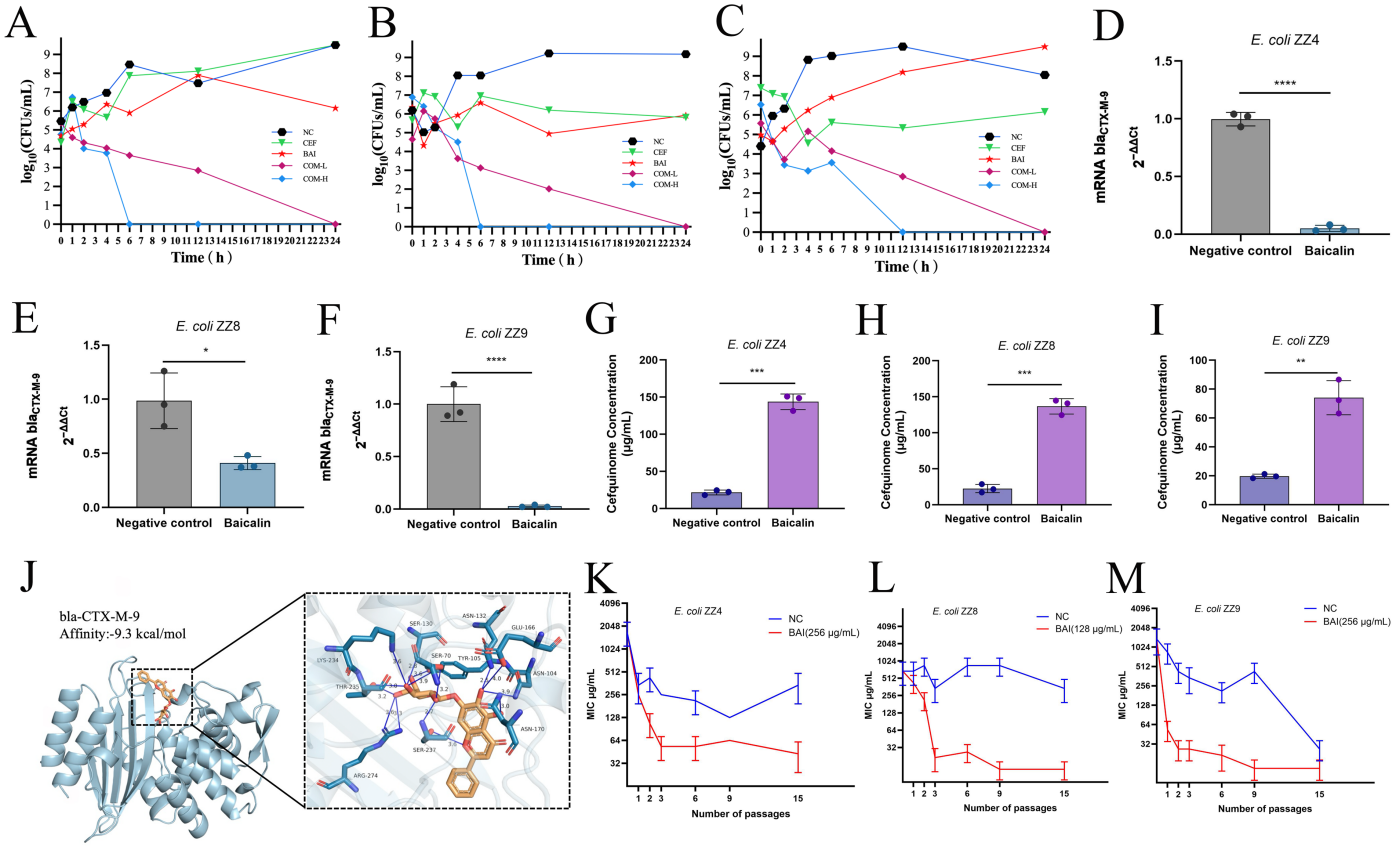
Baicalin (BAI) potentiates cefquinome (CEF) *in vitro* by attenuating resistance-associated phenotypes in ESBL-producing *E. coli*. **(A–C)** Time-kill curves of ESBL-*E. coli* ZZ8 treated with BAI, CEF, or their combinations at subinhibitory concentrations over 24 h. **(D–F)** Relative transcription levels of *blaCTX-M-9* in different treatments measured by qPCR. **(G–I)** Residual CEF concentrations in bacterial suspensions after hydrolysis assays with or without BAI. **(J)** Representative molecular docking pose of BAI within the CTX-M-9 β-lactamase catalytic pocket showing hydrogen bonds and hydrophobic contacts with key residues (Ser70, Lys73, Glu166). **(K–M)** Serial passage assay showing MIC changes of CEF over 15 passages in presence or absence of BAI. Data are mean ± SD (*n* = 3). Statistical significance determined using one-way ANOVA with Tukey’s post hoc test; *p* < 0.05 versus control.

Time-kill analysis showed that the BAI-CEF combination produced stronger bactericidal activity than either agent alone under subinhibitory concentrations, resulting in a marked reduction in viable bacterial counts over 24 h (Figures 4A–C). These results indicate that BAI potentiates CEF activity rather than acting as a strong standalone inhibitor under the tested conditions.

At the molecular level, qPCR analysis demonstrated that BAI significantly reduced transcription of *blaCTX-M-9* in ESBL-*E. coli*, with more pronounced suppression of *blaCTX-M-9* (Figures 4D–F).

CEF hydrolysis assays showed that BAI reduced ESBL-associated CEF hydrolysis in whole bacterial suspensions (Figures 4G–I). In BAI-treated bacterial suspensions, the CEF concentration decreased from the initial 200 μg/mL to 90–150 μg/mL, significantly higher than in the control group (20–30 μg/mL). These findings show that BAI reduces ESBL-mediated cefquinome hydrolysis in whole bacterial suspensions, suggesting interference with *β*-lactamase-related phenotypes, but direct inhibition must be assessed using purified enzymes.

Molecular docking further supported this interaction. The three-dimensional structure of BAI was docked into the catalytic pocket of CTX-M-9 β-lactamase using AutoDock Vina. The docking pose with the lowest binding energy revealed that BAI formed multiple hydrogen bonds and hydrophobic contacts with key active-site residues, including Ser70, Lys73, and Glu166, suggesting potential steric hindrance and stabilization within the enzyme’s catalytic site. Although this computational evidence does not definitively prove enzymatic inhibition, it provides mechanistic support for the observed reduction in CEF hydrolysis and resistance-associated phenotypes (Figure 4J).

Finally, serial passage experiments demonstrated that continuous exposure to subinhibitory BAI constrained the progressive increase in CEF MIC over 15 passages, resulting in a 2- to 32-fold reduction relative to the control (Figures 4K–M). These findings suggest that BAI can limit the development of reduced CEF susceptibility during repeated exposure.

Collectively, these *in vitro* results indicate that BAI potentiates CEF activity against ESBL-*E. coli* by enhancing bactericidal efficacy, suppressing ESBL-mediated antibiotic hydrolysis, and constraining resistance development, providing mechanistic support for the enhanced *in vivo* therapeutic effect observed with the combination treatment.

## 4 Discussion

The present study identifies BAI as a CEF sensitizer against animal-origin extended-spectrum beta-lactamase ESBL-*E. coli*. Mechanistically, this sensitization effect appears to emerge from a coordinated interaction between suppression of bacterial resistance determinants and improvement of the infection-altered intestinal microenvironment, which together amplify the effective antibacterial activity of CEF in vivo. The key finding is that BAI improved CEF performance through two coordinated dimensions: attenuation of bacterial resistance-associated phenotypes and mitigation of infection-induced intestinal injury. This dual-action profile is important in veterinary anti-infective therapy, where treatment failure often reflects not only reduced antibiotic susceptibility, but also persistent inflammatory and structural damage within the infected intestinal environment (30, 31).

At the pathogen level, BAI attenuated several resistance-associated phenotypes that are likely to limit CEF activity. BAI reduced *blaCTX-M* transcription, suggesting that it suppresses resistance-related gene expression rather than merely acting downstream of existing enzyme activity. Bacterial suspensions pretreated with BAI retained higher residual CEF concentrations, indicating reduced antibiotic inactivation. This functional reduction is consistent with the transcriptional suppression of blaCTX-M and provides a phenotypic bridge between gene-level regulation and antibiotic susceptibility. Beyond immediate enzymatic effects, such regulatory and functional changes may also influence long-term adaptive trajectories under antibiotic pressure. Serial passage experiments showed that BAI constrained the upward drift of CEF MIC over time, suggesting a suppressive effect on the evolution of reduced susceptibility. Taken together, these findings indicate that BAI acts on multiple phenotypic layers relevant to beta-lactam resistance and bacterial survival, thereby increasing the effective antibacterial pressure exerted by CEF. Among these pathogen-directed effects, the beta-lactamase-related findings are particularly relevant (32, 33). Collectively, these multi-level observations establish a convergent line of evidence linking transcriptional regulation, functional enzyme activity, and structural compatibility at the catalytic site. These observations suggest that BAI may not act as a direct enzymatic inhibitor, but rather influences resistance phenotypes at a regulatory level, although the precise upstream genetic or plasmid-associated mechanisms remain to be clarified. The combination of reduced *blaCTX-M* transcription, decreased hydrolysis of CEF, and favorable docking of BAI within the CTX-M-9 catalytic pocket supports the hypothesis that BAI interferes with ESBL-associated antibiotic inactivation. It should be noted that the docking result provides supportive rather than definitive evidence, and the hydrolysis assay was performed at the level of bacterial suspensions rather than purified enzyme kinetics. Accordingly, the present data are best understood as showing that BAI attenuates ESBL-associated phenotypes in a functionally meaningful way, rather than conclusively establishing direct enzyme inhibition. In this context, the combined evidence supports a multi-layer modulation model rather than a single-target inhibition mechanism. Even with this conservative interpretation, the observed effect remains mechanistically and therapeutically significant. The resistance-development experiment further strengthens the translational relevance of this study. By constraining the increase in CEF MIC during serial passage, BAI demonstrated a resistance-suppressive effect that extends beyond immediate growth inhibition. This feature is particularly valuable in the context of ESBL-*E. coli* infection, where recurrent or prolonged antibiotic exposure is a major driver of treatment failure (34–37).

The host-directed findings are equally important ESBL-*E. coli* challenge triggered marked intestinal injury, inflammatory activation, and disruption of mucosal architecture (38, 39). Importantly, these host responses are not independent events, but are closely coupled to pathogen burden and microbial activity within the intestinal niche. These changes were only partially improved by CEF alone, whereas BAI-containing regimens produced broader protection. This suggests that therapeutic efficacy is driven not only by direct antibacterial activity, but also by modulation of host-pathogen interaction dynamics. Specifically, the combination reduced jejunal expression of *Tlr4*, *Myd88*, and NF-κB-related inflammatory genes, improved villus architecture, preserved goblet cell abundance, and reduced inflammatory cell infiltration. These observations indicate that BAI contributes to therapeutic efficacy not simply by reducing bacterial burden, but also by limiting host tissue damage that sustains disease severity. Preservation of epithelial structure and local barrier function is critical for intestinal recovery, not only bacterial killing. An additional point of interest is that the low-dose combination appeared to provide a more balanced intestinal protective effect than the high-dose combination, despite the latter showing the most complete survival benefit. Such a divergence indicates that therapeutic outcomes are shaped by a balance between antimicrobial pressure and host tissue tolerance. This divergence suggests that optimal host protection and maximal short-term survival are not necessarily achieved at the same exposure combination. One plausible explanation is that the lower-dose regimen was sufficient to reduce bacterial burden while placing less physiological stress on the already damaged intestinal environment, thereby allowing better preservation of mucosal architecture. This observation highlights the importance of considering host tissue response, and not only survival or antibacterial potency, when evaluating antibiotic sensitizer strategies (40, 41). Although the precise immunological mechanisms remain to be fully defined, the observed changes in sIgA likely reflect shifts in antigenic stimulation and mucosal immune feedback. Therefore, sIgA alterations should be interpreted as indicators of mucosal immune status rather than direct evidence of immune enhancement or resolution of infection. The sIgA findings further support a context-dependent host-directed action of BAI. Infection increased ileal sIgA, consistent with activation of mucosal immune defense under bacterial challenge. In the combination groups, the reduction in sIgA may reflect changes in infection-associated mucosal stimulation after treatment, whereas the higher sIgA level observed with BAI monotherapy indicates a distinct mucosal immune response pattern. However, these interpretations remain descriptive and require further cellular and cytokine-level validation.

The microbiota data add another layer to this host-protective profile. Given the close bidirectional relationship between intestinal inflammation and microbial composition, these microbial shifts should be interpreted as part of an integrated host–microbiota response rather than isolated taxonomic changes. ESBL-*E. coli* challenge was associated with a shift in intestinal community structure, whereas BAI treatment partially moved the microbiota toward a configuration closer to that of uninfected controls. These findings should be interpreted conservatively: they support partial restoration of microbiota homeostasis at the community level, but do not justify strong functional claims about individual taxa (42, 43). The results are best viewed as evidence that BAI influences the intestinal ecological environment during infection, rather than as direct proof that specific taxa mediate therapeutic effect.

Taken together, these findings support a proposed mechanistic framework in which BAI enhances CEF efficacy through upstream suppression of resistance-associated determinants, leading to reduced antibiotic inactivation and slower resistance evolution, while simultaneously improving the intestinal microenvironment, thereby collectively amplifying *in vivo* therapeutic outcomes.

Several limitations should be acknowledged. The study was based on a limited number of ESBL-producing isolates, and thus may not fully capture the genetic diversity of clinical strains. Mechanistic interpretation of *β*-lactamase-related effects was based on transcriptional and phenotypic evidence, without protein-level validation or purified enzyme kinetics. In addition, microbiota analysis was restricted to taxonomic resolution due to sample size constraints. The present mechanistic framework is derived from pharmacodynamic and phenotypic observations, without direct pharmacokinetic characterization of baicalin or cefquinome in vivo. Therefore, potential influences of systemic exposure, tissue distribution, and local intestinal concentrations on their interaction dynamics cannot be excluded. These limitations do not negate the main conclusions, but they define the boundaries within which the results should be interpreted.

In conclusion, BAI is a promising CEF sensitizer against animal-origin ESBL-*E. coli*. Its therapeutic value arises from a dual mechanism that combines attenuation of resistance-associated bacterial phenotypes with protection of intestinal integrity, providing a rationale for the development of BAI-based sensitizer strategies in veterinary anti-infective therapy.

## 5 Conclusion

In conclusion, BAI enhanced the therapeutic performance of CEF against animal-origin ESBL-*E. coli* by combining pathogen-directed sensitization with host-directed intestinal protection. This effect was associated with reduced resistance-related phenotypes, attenuated inflammatory and structural intestinal injury, and partial normalization of intestinal microbiota structure. These findings support BAI as a promising veterinary antibiotic adjuvant candidate and provide a basis for further validation in clinically relevant infection models.

## Data Availability

The data presented in the study are deposited in the NCBI repository, accession number: PRJNA1498785, https://www.ncbi.nlm.nih.gov/bioproject/PRJNA1498785/.
